# Correlations between *ACE* single nucleotide polymorphisms and prognosis of patients with septic shock

**DOI:** 10.1042/BSR20170145

**Published:** 2017-04-28

**Authors:** Xin-Man Dou, Hui-Juan Cheng, Ling Meng, Lin-Lin Zhou, Yi-Hong Ke, Li-Ping Liu, Yu-Min Li

**Affiliations:** 1 Life Sciences School of Lanzhou University, Lanzhou 730000, P.R. China; 2 Department of General Surgery, Second Hospital of Lanzhou University, Lanzhou 730030, P.R. China; 3 Key Laboratory of Digestive System Tumors of Gansu Province, Lanzhou 730030, P.R. China; 4 Nursing School of Lanzhou University, Lanzhou 730000, P.R. China; 5 Department of Intensive Care Unit, First Hospital of Lanzhou University, Lanzhou 730030, P.R. China

**Keywords:** Angiotensin I-converting enzyme, Prognosis, Septic shock, Single nucleotide polymorphisms

## Abstract

The aim of the present study is to investigate association between septic shock (SS) and angiotensin I-converting enzyme (*ACE*) single nucleotide polymorphisms (SNPs). From October 2009 to December 2016, 238 SS patients and 242 healthy individuals were selected for our study. ACE activity was detected, *ACE* rs4291 and rs4646994 polymorphisms were detected using PCR-restriction fragment length polymorphism (PCR-RFLP). The Kaplan–Meier survival curve was employed to evaluate the association between *ACE* SNPs and patients’ survival and univariate and multivariate analyses to estimate risk factors for SS. ACE activity in the case group was increased in comparison with the control group. Allele and genotype frequencies of rs4291 and rs4646994 were different between the case and control groups. The TT genotype frequency of the rs4291 polymorphisms and the DD genotype of the rs4646994 polymorphisms of the case group were higher than those in the control group. The AT and TT genotypes indicated a significant elevation of ACE activity than the AA genotype, while a significant decline was found in the DI and II genotypes in comparison with the DI genotype. Patients with TT or DD genotypes had increased fatality rate within 7 and 30 days when compared with those with non-TT or non-DD genotypes. Lower sepsis-related organ failure assessment (SOFA) scores, rs4291, serum ACE and rs4646994 were all considered as risky factors for SS patients. The study demonstrates that TT genotype of rs4291 or DD genotype of rs4646994 may be indicative of a higher risk of SS and a poorer prognosis in SS patients.

## Introduction

Sepsis is determined by the endogeneous glucocorticoids [1], resulting in multiorgan failure, septic shock (SS) and even death if not diagnosed earlier or treated promptly [2]. Unfortunately, it is currently still grossly under-recognized in the world. SS is a severe systemic reaction to bacterial infection [3]. The occurrence of sepsis, severe sepsis and SS has been on the rise, where SS and severe sepsis cause frequent complications and are the leading causes of death for patients in intensive care unit (ICU) in China [4–6]. Severe sepsis and SS lead to 215000 deaths annually in U.S.A. [7]. Though treatment for severe sepsis and SS has been positively affected by clinical research studies conducted over the past 10 years, challenges still remain with regard to the early haemodynamic optimization [8]. Interestingly, poor prognosis (up to 50% mortality rate) still happens in SS patients, though substantial progress has been made in both fundamental and clinical studies [9]. Antibiotic therapy is reported to be functional not only in improving clinical response, but also the survival of life-threatening infections, especially those correlated with SS; studies have revealed that impaired prognosis is followed by delayed antibiotic therapy [10,11]. Various gene polymorphisms have been proved to be linked with the development of SS and severe sepsis, such as the tumor necrosis factor (*TNF*) gene, but some may also be associated with the increased mortality rate in SS patients like the *ADRB2* gene [12,13].

Angiotensin I-converting enzyme (*ACE*) gene polymorphisms have been correlated with susceptibility to multiple inflammatory diseases, including psoriasis and systemic lupus erythematosus; furthermore, angiotensin II (AngII) pathway is known to play a vital part in SS [14]. Previous reports indicate that ACE, as a central enzyme in the renin–angiotensin–aldosterone system, exerts a crucial role in the development of cardiovascular system diseases [15] with the insertion/deletion (I/D) polymorphism. A strong relevance is also suggested between *ACE* I/D gene polymorphism and gestational diabetes mellitus among Asian Indian women [16]. Moreover, the *ACE* gene, with the absence of the I allele has been related to higher ACE circulation levels as well as lower hypertension. Additionally, the *ACE* genotypes may play a role in the release of nitric oxide and blood pressure after exercise [17]. The *ACE* polymorphisms rs4291 and rs1800764 have indicated a relationship with the risk of late-onset Alzheimer’s disease (LOAD) and haplotypes of *ACE* are connected with ACE level in plasma and LOAD risk [18]. In addition, some single nucleotide polymorphisms (SNPs) in the *ACE* gene are correlated with increased 4-week mortality of SS, resulting in a poor prognosis [14]. With the aim to improve the prognosis of SS and provide help in the evaluation of therapies for SS patients, the present study was conducted to investigate the correlation between rs4291 and rs4646994 of the *ACE* gene and the prognosis of SS.

## Materials and methods

### Study subjects

From October 2009 to December 2016, 238 patients diagnosed with SS (known as the case group) and 242 healthy individuals (known as the control group) were recruited for our study. All the included subjects were in accordance with the definition and requirements for sepsis developed at the 2001 SCCM/ESICM/ACCP/ATS/SIS International Sepsis Definitions Conference, sponsored by the Society of Critical Care Medicine (SCCM), European Society of Intensive Care Medicine (ESICM), American College of Chest Physicians (ACCP), American Thoracic Society (ATS) and Surgical Infection Society (SIS) [19]. Patients with any of the following conditions were excluded: pregnant, below 18 years of age, chronic organ failure, susceptibility to tumour, immune deficiency, blood transfusion within half a month, bone marrow transplantation or acute poisoning in 15 days. The severity of illness was classified by acute physiology and chronic health evaluation II (APACHE II) and sepsis-related organ failure assessment (SOFA) scores. Treatment was in strict accordance with the guidelines for sepsis bundles in 2006 and all collected subjects were recruited from Chinese Han population. Clinical observation started 30 days after patients were admitted into the intensive care unit (ICU) and detailed clinical characteristics of patients were collected, including the source of microbial infection, APACHE II score after 24-h ICU admission, worst SOFA score after 7-day of ICU admission, shock period and survival after 7 and 30 days of ICU admission. The present study was approved by the Ethics Committee of Second Hospital of Lanzhou University and all the study subjects provided signed informed consents.

### Blood sampling and DNA extraction

Peripheral venous blood samples (5 ml) were collected from the patients in the case group within 24 h of admission while those of the control group were collected during physical examination, and the samples were added with 2% EDTA. Full blood amount (200 μl) was used for DNA extraction using a QIAamp DNA Blood Mini Kit (Qiagen, Hilden, Germany) and DNA content was determined by a UV spectrophotometer with the A_260_/A_280_ ratio in the range of 1.8–2.0. The required DNA template concentration for PCR was calculated. Extracted genomic DNA was stored in the TE (Tris-EDTA) buffer at –80°C before analysis.

### *ACE* activity

A total of 3 ml of blood was extracted on an empty stomach, with serum separated by centrifugation (3000 rev/min) at 57°C after 30 min. The HITACHI-7170 Auto-biochemical Analyzer was used for ACE reagent to detect the ACE activity.

### Detection of SNPs in *ACE*

**Figure 1:**
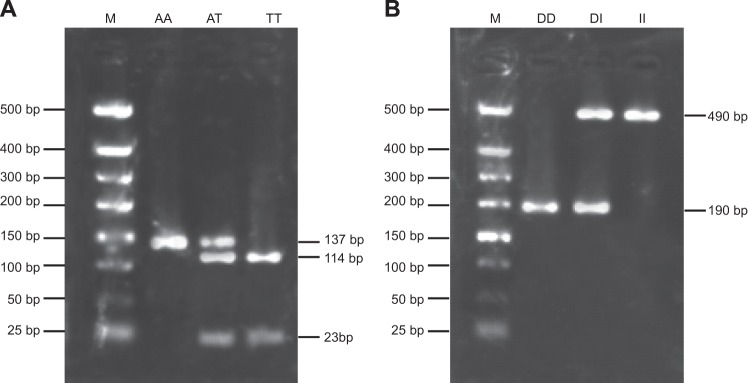
Enzyme electrophoresis of *ACE* rs4291/rs4646994 (**A**) Enzyme electrophoresis of rs4291; (**B**) enzyme electrophoresis of rs4646994; M, marker.

The SNPs of *ACE* gene rs4291/rs4646994 were selected and the sequences of *ACE* were obtained from the Gene Bank. Primer Premier 5.0 software was applied to design the PCR primers. The genotypes rs4291 and rs4646994 were amplified in the same fragment due to their close distance. The primer sequences used were as follows: rs4291: 5′-ACGTTGGATGGCAGAGGAAGCTGGAGAAAG-3′ (forward); 5′-ACGTTGGATGTCGGGTGTTCCGGCAAACTG-3′ (reverse); rs4646994: 5′-CTGGAGACCACTCCCATCCTTTCT-3′ (forward); 5′-GATGTGGCCATCACATTCGTCAGAT-3′ (reverse). The total volume of reaction was 10 μl, including 5 μl of DNA (5 ng/pl), 1 μl of 10× buffer solution, 1.5 μl of primer for each group, 0.4 μl of 2′-deoxyribonucleoside triphosphates (dNTPs), 0.5 μl of (2.5 U) Taq DNA polymerase, 0.5 μl of ddH_2_O. A gradient PCR instrument was used and the amplification conditions were as follows: 3 min of pre-denaturation at 94°C, 35 cycles of denaturation for 45 s at 94°C, annealing for 45 s at 58°C and extension for 60 s at 72°C and a final extension for 7 min at 72°C and the PCR products were stored at 4°C. A total of 5 μl of PCR products were detected and analysed by 2.5% agarose gel electrophoresis. After successful amplification, Hha I endonuclease for rs4291 and Tai I for rs4646994 were added respectively and kept in an incubator at 37°C for 4–16 h, followed by an analysis regarding the restriction map using gel imaging system, and the genotypes were detected after 30-min 2.5% agarose gel electrophoresis (Figure 1).

### Statistical methods

SPSS 21.0 software (SPSS Inc., Chicago, IL, U.S.A.) was applied for data analysis. Measurement data were expressed as mean ± S.D. and enumeration data as ratio, while deviations from Hardy–Weinberg equilibrium (HWE) were estimated as well. Odds ratio (OR) with 95% confidence interval (95% CI) was used to estimate the risk of allele. SOFA and APACHE II scores were compared by Kruskal–Wallis non-parametric test, and the Kaplan–Meier method was performed to analyse the association of genotypes of *ACE* with the survival of patients with SS. Univariate and multivariate analysis was used for risky factors for SS. Chi-square test or Fisher’s exact test was conducted to compare categorical variables. The genotypes and allele frequencies and haplotypes analyses were performed by the Shesis software. The level of significant difference was set as two-tailed *P*<0.05.

## Results

### Baseline characteristics of the subjects in the case and control groups

The case group consisted of 133 males and 105 females with a mean age of 52.09 ± 7.06 years. APACHE II score, worst SOFA score after 7-day ICU admission and infectious characteristics in the 30-day observation are as shown in Table 1. The control group consisted of 128 males and 114 females with a mean age of 52.96 ± 5.32 years. The case group indicated significantly increased ACE activity in comparison with the control group (*P*<0.05).

**Table 1:** Baseline characteristics of patients with SS

Characteristic	Case group (*n*=238)	Control group (*n*=242)	*P*
Age (years)	52.09 ± 7.06	52.96 ± 5.32	0.128
Gender (male/female)	133/105	128/114	0.511
APACHE II	32.10 ± 12.37		
SOFA	9.02 ± 2.77		
Source of infection (*n* (%))			
Lung	73 (43.28)		
Abdomen	53 (24.37)		
Peripheral blood	49 (30.67)		
Wounds	28 (14.71)		
Other parts	35 (15.97)		
Infectious bacteria			
G^+^ bacteria	69 (43.70)		
G^−^ bacteria	93 (74.37)		
Mixed infection	55 (28.57)		
Unknown infection	21 (10.08)		
ACE activity (U/l)	53.59 ± 6.48	46.81 ± 5.92	< 0.001

### HWE

Both the case and the control groups received a χ^2^ goodness-of-fit test for HWE in the distribution of genotype frequencies of rs4291/rs4646994 of the subjects. The results demonstrated an approximately identical observation number to the expectation number, indicating that the genotype frequencies of rs4291/rs4646994 were in accordance with the HWE (*P*>0.05) and demonstrated good representativeness (Table 2).

**Table 2:** HWE test of two *ACE* SNPs

SNP	Genotype	Case group				Control group			
		O	E	χ^2^	*P*	O	E	χ^2^	*P*
rs4291				0.102	0.95			1.385	0.5
	AA	59	60			105	99		
	AT	122	119			100	112		
	TT	57	59			37	31		
				0.308	0.857			0.843	0.656
rs4646994	DD	52	49			35	32		
	DI	112	118			102	112		
	II	74	71			105	98		

E, expected number; O, observed number.

### Comparison of the genotype and allele frequencies of *ACE* rs4291/rs4646994

Results of the χ^2^ goodness-of-fit test indicated that the genotype and allele frequencies of rs4291 and AT genotype in the case group were of significant difference, in comparison with those in the control group (all *P*<0.05). Frequencies of TT genotype (49.58% compared with 35.95%) and T allele (49.58% compared with 35.95%) were remarkably higher, while A allele (50.42% compared with 64.05%) frequency was significantly lower in the case group than those in the control group (all *P*<0.05). Genotype and allele frequencies of rs4646994 and DI genotype in the case group were evidently different when compared with those in the control group (all *P*<0.05). Frequencies of DD genotype (21.85% compared with 14.46%) and D allele (45.37% compared with 35.53%) in the case group were remarkably higher, and I allele (54.63% compared with 64.47%) frequency was remarkably lower than that in the control group (all *P*<0.05) (Table 3).

**Table 3:** Frequencies of genotypes and alleles of two *ACE* SNPs in the case and control groups

Genotype/allele	Control group	Case group	χ^2^	*P*
	(*n*=242)	(*n*=238)		
rs4291				
AA	105 (43.39)	59 (24.79)		Ref.
AT	100 (41.32)	122 (51.26)	13.64	<0.001
TT	37 (15.29)	57 (23.95)	14.69	<0.001
A	310 (64.05)	240 (50.42)		Ref.
T	174 (35.95)	236 (49.58)	18.22	<0.001
rs4646994				
DD	35 (14.46)	52 (21.85)	7.976	0.005
DI	102 (42.80)	112 (47.06)	4.727	0.03
II	105 (43.39)	74 (31.09)		Ref.
D	172 (35.53)	216 (45.37)	9.651	0.002
I	312 (64.47)	260 (54.63)		Ref.

Ref., reference, i.e. AA/II genotype as the controls.

### Correlation of *ACE* polymorphism and ACE activity

The correlation of *ACE* polymorphisms, rs4291 and rs4646994, as well as *ACE* activity are as shown in Table 4. A significant elevation in ACE activity is demonstrated in the AT and TT genotypes (TT > AT) compared with the AA genotype and a significant decline in the DI and II genotype (DI > II) compared with the DI genotype.

**Table 4:** Correlation of two *ACE* SNPs and *ACE* activity

Genotype/allele	ACE activity (U/l)	*P*
rs4291		
AA	49.75 ± 5.47	
AT	53.85 ± 5.18	<0.001
TT	57.00 ± 7.81	<0.001
rs4646994		
DD	60.05 ± 5.06	
DI	53.27 ± 5.45	<0.001
II	49.53 ± 5.17	<0.001

### Correlation between the genotype and allele frequencies of *ACE* and the survival condition

According to the prognosis, a total of 238 patients with SS were divided into the death group (*n*=73) and the survival group (*n*=165). The frequencies of rs4291 TT and rs4646994 DD genotypes were significantly higher in the death group than that in the survival group. In rs4291, the frequency of T allele in the death group was 72.6%, higher than the 39.39% in the survival group and the frequency of A allele (27.40%) was lower than that in the survival group (60.61%, both *P*<0.05). In rs4646994, the frequency of D allele in the death group (65.07%) was higher than that in the survival group (36.67%) and the frequency of I allele was 34.93%, lower than the 63.33% in the survival group (both *P*<0.05) (Table 5).

**Table 5:** Frequencies of genotypes and alleles of two *ACE* SNPs in the death and survival groups

Genotype/allele	Survival group	Death group	χ^2^	*P*
	(*n*=165, 69.33%)	(*n*=73, 30.67%)		
rs4291				
AA	57 (34.55)	2 (7.69)		Ref.
AT	86 (52.12)	36 (49.31)	16.36	<0.001
TT	22 (13.33)	35 (34.62)	44.92	<0.001
A	200 (60.61)	40 (27.40)		Ref.
T	130 (39.39)	106 (72.60)	44.65	<0.001
rs4646994				
DD	20 (12.12)	32 (43.84)	31.7	<0.001
DI	81 (49.10)	31 (42.47)	5.2	0.023
II	64 (38.78)	10 (13.69)		Ref.
D	121 (36.67)	95 (65.07)	32.94	<0.001
I	209 (63.33)	51 (34.93)		Ref.

Ref., reference, i.e. A/I as the controls.

### Haplotype analysis

Haplotypes of *ACE* rs4646994/rs4291 (Table 6) in the case and control groups were analysed by a software (haplotype with a frequency over 0.03 was excluded). The results revealed that the frequencies of AI, TD and AD haplotypes were of difference in the two groups (all *P*<0.05). TD haplotype was the risk factor for SS, while AI and AD haplotypes were protective factors for SS. The frequency of TI haplotype had no difference in the two groups (both *P*>0.05).

**Table 6:** Haplotype analysis of *ACE* rs4646994 and rs4291 polymorphisms

Haplotype		Case group	Control group	*P*	OR	95% CI
A	I	130 (0.273)	166 (0.344)	0.017	0.715	0.543-0.942
A	D	110 (0.231)	143 (0.296)	0.022	0.714	0.535-0.954
T	I	130 (0.273)	145 (0.300)	0.352	0.875	0.662-1.158
T	D	106 (0.223)	28 (0.059)	< 0.001	4.557	2.949-7.041

### Prognosis of SS patients with different genotypes of *ACE*

**Figure 2:**
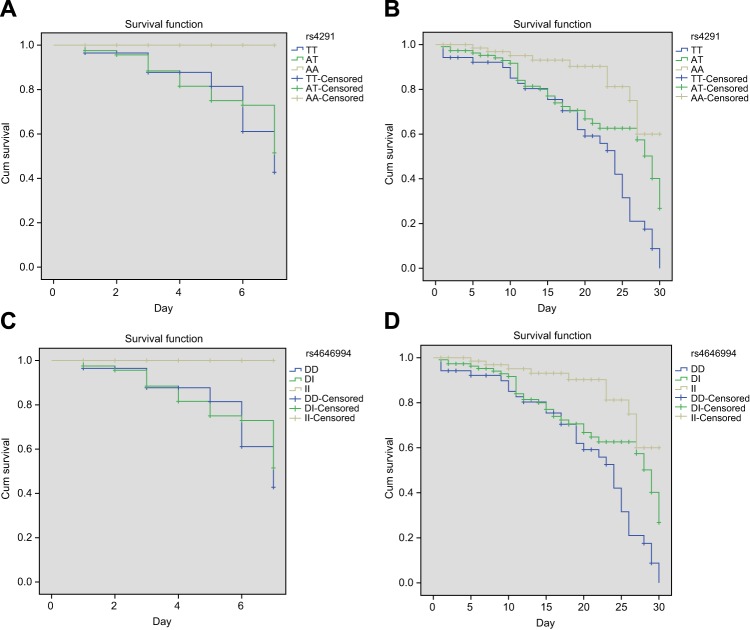
Kaplan–Meier survival curve for 7- and 30-day mortality of patients with rs4291 (**A** and **B**) and rs4646994 (**C** and **D**) (**A** and **C**) 7-day survival curves; (**B** and **D**) 30-day survival curves.

One month clinical follow-up was conducted on 238 patients and 73 patients died of SS during this period. The 7 and 30 days mortality rates were 17.65 and 30.67% respectively. The Kaplan–Meier method was used to analyse the correlations between different genotypes of rs4291/rs4646994 as well as the 7- and 30-day prognoses. The results demonstrated that different genotypes of rs4291/rs4646994 indicated significantly different survival curves. In comparison with the patients without rs4291 TT or rs4646994 DD genotypes, patients with these genotypes had much higher mortality rate (*P*=0.005; *P*<0.001) (Figure 2).

### Univariate and multivariate analysis for risk factors for the prognosis of SS patients

Of 238 SS patients, 42 died in 7 days. High APACHE II, level of serum ACE and DD genotype of rs4646994 increased mortality rate, while low SOFA score and AA genotype of rs4291 could decrease mortality rate of SS (all *P*<0.05). The shock period was irrelevant (*P*>0.05). Regarding the 30 day mortality rate, the univariate analysis revealed that patients with longer shock period, high APACHE II, level of serum ACE and DD genotype of rs4646994 had elevated mortality rate, while low SOFA score AA genotype of rs4291 indicated a decrease in mortality rate, as shown in Table 7. The factors mentioned above were included in the multivariate analysis, the results indicated that there was a decrease in fatality rate in regard to low SOFA score and rs4291, while rs4646994 and high level of serum ACE, as risk factors for SS, indicated an increase fatality rate (all *P*<0.05) (Table 8).

**Table 7:** Univariate analysis for the risk factors for the 7- and 30-day mortality rates of patients with SS

Factor	7 Days			30 Days		
	Survival	Death	*P*	Survival	Death	*P*
Shock period	5.91 ± 2.18	6.40 ± 2.08	0.608	5.59 ± 2.09	6.91 ± 2.08	<0.001
APACHE II score	30.92 ± 12.26	37.62 ± 11.46	0.001	30.84 ± 12.63	34.97 ± 11.32	0.017
SOFA score	8.63 ± 2.72	10.84 ± 2.27	<0.001	8.39 ± 2.74	10.43 ± 2.29	<0.001
rs4291						
AA compared with AT/TT	59/ 137	0/42	0.018	57/108	2/71	<0.001
rs4646994						
DD compared with DI/II	29 /167	23/19	<0.001	20/145	32/41	<0.001
Serum ACE	52.70 ± 6.28	57.76 ± 5.78	<0.001	52.81 ± 6.22	55.36 ± 6.74	<0.001

**Table 8:** Multivariate analysis of the risk factors for mortality rates of patients with SS

Factor	β	S.E.M.	Wald	*P*	OR	95% CI
APACHE II score	0.008	0.015	0.134	0.714	1.006	0.976–1.036
SOFA score	–0.238	0.07	11.469	0.001	0.788	0.687–0.905
rs4291	–2.465	0.897	7.544	0.006	0.085	0.015–0.494
rs4646994	0.889	0.38	5.479	0.019	2.432	1.156–5.117
Serum ACE	0.07	0.031	5.065	0.024	1.073	1.009–1.140

## Discussion

As one of the major causes of mortality recognized worldwide, sepsis induces SS, organ dysfunction or failure and even death in the case of late treatment [2,5]. Severe sepsis is defined as the occurrence of sepsis plus multi-organ dysfunction induced by sepsis and SS is defined by the presence of severe sepsis along with dangerously low blood pressure [20]. Prognosis of SS patients after ICU admission has been attracting great attention in recent years [21–25]. Previous studies have suggested that gene polymorphisms possibly play a significant role in SS and sepsis [13,26,27]. The present study aimed to investigate the correlations between the prognosis of SS and the SNPs of *ACE* gene; as a result, the study indicated that the prognosis of patients with SS were associated with *ACE* SNPs rs4291 and rs4646994. Moreover, patients with rs4291 TT genotype and rs4646994 DD genotype were more susceptible to death caused by SS.

Initially, the findings in our research suggested that the frequencies of genotype and allele of *ACE* rs4291/rs4646994 indicated a significant difference between the case and control groups. Interestingly, a different study indicated that the I allele carriers were discovered to be more susceptible to developing sepsis, which is in consistency with our results demonstrating that I allele frequency was found to be notably lower in the control group than the case group [27]. A correlation between polymorphisms in *HLA-G* gene and the condition of severely ill patients with SS has been found [28], with evidence identifying that patients with severe sepsis due to pneumonia are more likely to suffer from SS when carrying the 4G allele of PAI-1 polymorphism and poor prognosis of septic patients has been related to the polymorphism of interleukin-1 receptor antagonist (ILRN) 2 [26,29]. Importantly, ACE, an important component of renin–angiotensin system (RAS) encoded by *ACE* gene, is known to exert a crucial role in regulating blood pressure [30]. Cardiac dysfunction, a severe and frequent complication of SS, results in the high mortality rate of sepsis [31]. Along with sepsis, it is in close association with inflammation as well as declined fatty acid oxidation [32]. Some reports have also indicated an association of *ACE* I/D gene polymorphisms with alterations in the *ACE* inhibitors’ effectiveness and *ACE* inhibition would reduce organ dysfunction in critically ill septic patients [33,34]. One study suggested that patients treated with ACE inhibitors might have decreased short- and long-term mortality [35]. All data above were in line with the result that serum ACE activity in the case group was remarkably elevated than that in the control group.

Additionally, rs4291 TT and rs4646994 DD genotypes’ frequencies in the death group were remarkably higher than that in the survival group according to the comparison results in our study. ACE was found in close correlation with chronic obstructive pulmonary disease, asthma and acute respiratory distress syndrome [36]. In recent years, a pivotal role of ACE2, ACE and their peptides were recognized during the inflammatory process such as glomerulonephritis, pulmonary hypertension, sepsis, lung injury, acute pancreatitis and also cardiac hypertrophy [37], while also being a key part of the renin–angiotensin system [38]. Interestingly, AngII infusion resulted in proteinuria-independent renal impair only in rats with genetically predetermined high level of ACE, while rats with low ACE were probably protected from the detrimental influence of AngII [39]. A correlation was also revealed between *ACE* rs4291 TT and the declining cognition in patients with LOAD [18,40]. Also, it is proven that in comparison with the lowest ACE activity in II genotype carriers, DD genotype of *ACE* is related to the highest systemic and renal ACE levels [30]. Evidence indicates significant association between the D allele or DD genotype of *ACE* and the risk for IgA nephropathy in Asian populations [41]. It was also found that *ACE* rs4646994 DD genotype was associated with higher risk for primary spontaneous intracerebral haemorrhage patients [42].

Furthermore, the data of our study indicated that the 7- and 30-day mortality rates increased in patients with rs4291 TT genotype and rs4646994 DD genotype, results confirmed using the Kaplan–Meier method. Although great improvements have been achieved, SS continues to be the leading cause of mortality in ICU all over the globe [43]. The increased protein expression of AngII type 1 receptor was associated with increased 28-day mortality in SS as well as decreased blood pressure [44]. As for the strong risk factors for the prognosis of SS, our findings demonstrated that the lower SOFA scores and rs4291 could decrease fatality rate and rs4646994 and higher serum ACE could increase fatality rate. SOFA scores are popularly used in the prediction of hospital mortality in critically ill patients, and the application of the two combined is proved to have a better predictive value in ICU admissions [45,46]. High SOFA scores generally indicate serious situation and poor prognosis.

In conclusion, the present study supported a significant role for the *ACE* gene polymorphisms in the prognosis of SS. Evidence was provided for the roles of the rs4291 and rs4646994 SNPs and other risk factors for SS prognosis. However, due to the limit in terms of sample size and ethnic group, our results need to be further assessed by additional well-designed studies involving larger sample size and diverse ethnic populations.

## Abbreviations

ACCP: American College of Chest Physicians
ACE: angiotensin I-converting enzyme
AngII: angiotensin II
APACHE II: acute physiology and chronic health evaluation II
ATS: American Thoracic Society
ESICM: European Society of Intensive Care Medicine
HWE: Hardy–Weinberg equilibrium
ICU: intensive care unit
I/D: insertion/deletion
LOAD: late-onset Alzheimer’s disease
OR: odds ratio
SCCM: Society of Critical Care Medicine
SIS: Surgical Infection Society
SOFA: sepsis-related organ failure assessment
SNP: single nucleotide polymorphism
SS: septic shock
95% CI: 95% confidence interval
